# Pathological Narcissism and Psychological Distress Among University Students: The Interplay of Family Dynamics, Trauma, and Health-Related Factors

**DOI:** 10.3390/healthcare14162470

**Published:** 2026-08-10

**Authors:** Silvana Krnić, Ana Jerončić, Varja Gaić Ðogaš

**Affiliations:** 1 Clinic for Psychiatry, University Hospital of Split, 21000 Split, Croatia; 2 Department of Psychological Medicine, School of Medicine, University of Split, 21000 Split, Croatia; varja.dogas@mefst.hr; 3 Department of Research in Biomedicine and Health, School of Medicine, University of Split, 21000 Split, Croatia; ana.jeroncic@mefst.hr

**Keywords:** pathological narcissism, psychological distress, university students, chronic fatigue, insomnia, trauma, family dynamics, mental health

## Abstract

**Highlights:**

**What are the main findings?**

- Pathological narcissism—encompassing both grandiose and vulnerable dimensions—was robustly associated with global psychological distress (r = 0.636) in a large student sample, with chronic fatigue emerging as the strongest somatic correlate across all narcissism models.
- Worse perceived family relationship quality was positively associated with higher narcissism scores. Trauma exposure, insomnia, prior psychological help-seeking, and faculty affiliation (Humanities and Social Sciences > Medicine) were also significantly associated with higher narcissism scores; over half of the sample (54%) met criteria for clinically significant psychopathology.

**What are the implications of the main findings?**

- University counseling services should screen for the co-occurrence of pathological narcissism and somatic exhaustion, recognizing that chronic fatigue may signal a physiological cost of sustained narcissistic self-regulation rather than a purely physical complaint.
- Faculties should implement destigmatization campaigns and mental health programs that address personality vulnerability given that a small percentage of students seek help. It is particularly worrying that only 1% of students will decide to seek help from faculty or teaching staff.

**Abstract:**

**Background/Objectives:** The mental health of university students is a global public health priority. While pathological narcissism is increasingly recognized as a source of significant psychological suffering, its developmental and somatic correlates remain insufficiently explored. This study examines the sociodemographic, familial, and health-related correlates of pathological narcissism and its association with global psychological distress. **Methods:** A cross-sectional study was conducted with 888 university students (M = 21.3 years; 78.5% female) recruited from three faculties—Humanities and Social Sciences, Civil Engineering, and Medicine. Participants completed the Pathological Narcissism Inventory (PNI), Brief Symptom Inventory (BSI), and sociodemographic, familial, and health-related characteristics, including trauma exposure. Multivariable linear regressions and hierarchical regression analyses were conducted. **Results:** Pathological narcissism was robustly and positively associated with global psychological distress (r = 0.636, *p* < 0.001). Multivariable regression models identified chronic fatigue as the strongest somatic correlate of narcissistic pathology, consistent with an ego-depletion account of self-regulatory failure. Additional significant correlates included trauma history, insomnia, and prior use of psychological services. Students from the Faculty of Humanities and Social Sciences reported significantly higher narcissism scores than medical students. Poorer perceived family relationship quality was associated with higher narcissism scores. The full models accounted for 14.4% to 16.9% of the variance in narcissistic dimensions. **Conclusions:** These cross-sectional findings suggest that pathological narcissism is not merely a personality trait but a clinically relevant construct deeply intertwined with psychological distress and somatic exhaustion. The strong association between narcissism and chronic fatigue points to a substantial physiological cost of sustained self-regulatory effort. Findings highlight the need for integrated screening protocols in university counseling services that account for somatic symptoms (fatigue, insomnia), traumatic experiences, and family dynamics, alongside efforts to destigmatize help-seeking, given that fewer than 1% of students turn to faculty for support.

## 1. Introduction

Mental health difficulties among university students represent a significant and growing public health concern. Large-scale surveys consistently report that between 30% and 50% of students meet criteria for at least one mental health disorder during their studies, with anxiety and depression being the most prevalent [1,2,3]. University enrollment coincides with a particularly sensitive developmental period—emerging adulthood—during which individuals navigate identity consolidation, separation from family, increased academic demands, and complex interpersonal environments [4]. These stressors converge with a heightened biological susceptibility to the onset of affective and personality psychopathology, rendering this population a priority target for mental health screening and early intervention [5,6].

Recent studies have highlighted a rising prevalence of personality pathology and its complex relationship with global symptom burden. Among personality-level risk factors, pathological narcissism has emerged as a clinically relevant but systematically under-screened dimension in student populations [7,8]. This is notable given that narcissistic traits peak in young adulthood, are particularly sensitive to the identity challenges and social comparison pressures characteristic of the university environment and have been associated with a range of psychological difficulties spanning both internalizing and externalizing domains [9,10].

Debates about whether narcissism in the student population is on the rise have been ongoing for decades [11]. The methodologically most rigorous contribution to this question—a meta-analysis encompassing 1105 studies using the Narcissistic Personality Inventory—found that year of data collection was negatively associated with narcissism scores across nearly all analyses, indicating a decline rather than an increase over time. These findings effectively refuted the theory that a narcissism epidemic ever occurred [12].

Pathological narcissism and narcissistic personality disorder (NPD) are related but non-identical constructs. NPD is a categorical clinical diagnosis defined in the DSM-5 by a fixed set of criteria (e.g., grandiosity, need for admiration, lack of empathy) and applies to a comparatively small proportion of individuals who meet the full diagnostic threshold [12]. Pathological narcissism, by contrast, is a dimensional construct capturing the degree of narcissistic dysregulation—spanning both grandiose and vulnerable presentations—that can be present at clinically meaningful levels without meeting full criteria for NPD [13]. The Pathological Narcissism Inventory (PNI) was specifically developed to capture both the grandiose and vulnerable dimensions of pathological narcissism [14]. This distinction matters empirically: NPD, as traditionally conceptualized around overt grandiosity, has often been associated with poorer insight and treatment resistance [15], whereas pathological narcissism assessed dimensionally—particularly its vulnerable component—shows more consistent associations with subjective distress, help-seeking, and internalizing symptoms [16,17,18]. The present study concerns pathological narcissism as a dimensional trait, not NPD as a categorical diagnosis.

Contemporary models distinguish at least two distinct but interrelated dimensions of pathological narcissism. Grandiose narcissism is characterized by overt self-aggrandizement, a stable sense of superiority, extraversion, and a need for admiration, and has been associated with relative psychological resilience and positive affective experience at the trait level [12,13,14]. Vulnerable narcissism, by contrast, reflects a covert, shame-based form of narcissistic self-organization marked by hypersensitivity to criticism, affective instability, contingent self-esteem, and chronic interpersonal distress [8,15].

Extensive psychological literature emphasizes that the mental health correlates of narcissism heavily depend on whether it manifests as a personality trait or as a pathological state, as well as on the specific instruments used for assessment. Non-clinical, trait-level grandiose narcissism—commonly measured using the Narcissistic Personality Inventory (NPI)—frequently demonstrates a positive association with indicators of subjective well-being, social self-esteem, and short-term adaptive resilience [16]. In contrast, vulnerable narcissism is consistently and strongly linked to negative mental health outcomes, such as anxiety, depression, and severe emotional dysregulation [17].

Neurobiological research on HPA axis reactivity has documented markedly distinct patterns of stress-related activation across narcissistic subtypes [18], while the association between pathological narcissism and both distress and burnout appear to be mediated by cognitive processes such as fear of failure [19].

The highly demanding environment of medical education constitutes a specific context for the expression of narcissistic vulnerability. Although academic burnout rates in this population are extraordinarily high—ranging from 5.6% to 88% across studies—the role of personality structure in generating that distress has remained largely overlooked [20,21,22,23,24,25,26,27,28,29].

A growing body of evidence implicates developmental and health-related factors in the expression of narcissistic pathology. Trauma exposure, particularly emotional maltreatment and neglect during childhood, has been identified as a robust correlate of vulnerable narcissism, operating in part through disruptions to attachment security [28,29]. The emotional hyperactivation characteristic of narcissistic vulnerability imposes sustained demands on psychophysiological regulatory systems and may manifest in somatic complaints [15,30].

Research on pathological narcissism in student populations in Croatia remains limited. To our knowledge, only one study has examined pathological narcissism—using a multidimensional instrument distinguishing grandiose and vulnerable dimensions—as a correlate of psychological distress in this specific population, and it was published by the present authors [31]. This represents a meaningful gap, given the documented cultural specificity of narcissistic trait expression, the relatively collectivistic orientation of Croatian society, and the substantial variability in student mental health across European national contexts [10,30].

The present study therefore examined associations between pathological narcissistic traits—as measured by the PNI—and clinically significant psychological distress, measured by the BSI, among students from three faculties of the University of Split. We further explored the role of family dynamics and somatic health indicators, particularly chronic fatigue and insomnia, as correlates of narcissistic pathology, and examined differences in narcissistic traits across faculties, genders, and academic years.

Given the relatively limited prior research jointly examining pathological narcissism, family dynamics, and somatic health indicators in student populations, and the absence of prior investigations within this specific sociocultural context, the present study adopted an exploratory approach. Rather than testing pre-specified directional hypotheses, we aimed to broadly characterize associations between pathological narcissistic traits and clinically significant psychological distress, as well as potential differences across faculties, genders, and academic years. Of particular interest was the role of family dynamics and somatic health indicators—specifically chronic fatigue and insomnia—as correlates of narcissistic pathology. By identifying candidate vulnerability factors associated with pathological narcissistic traits and distress, this study aims to advance a comprehensive, hypothesis-generating understanding of student well-being and inform institutional interventions designed to strengthen mental health support within higher education.

## 2. Materials and Methods

### 2.1. Design

This cross-sectional study included 888 students from three faculties at the University of Split, Croatia. The target population comprised all students enrolled in the first, third, and final (fifth or sixth) academic years who were present during mandatory class sessions. In terms of academic affiliation, the sample was deliberately drawn from three faculties—the Faculty of Humanities and Social Sciences, the Faculty of Civil Engineering, Architecture, and Geodesy, and the School of Medicine—to enable comparison across differing academic environments.

### 2.2. Sample Size Estimation

The minimum sample size was calculated based on precision estimates. To achieve a margin of error of ±5% at a 95% confidence level for proportions within the student population, a minimum of 381 participants was required, as determined using MedCalc^®^ Statistical Software version 23.0.6 (MedCalc^®^ Software Ltd., Ostend, Belgium). This calculation assumed a response distribution of 50%, which provided the most conservative sample estimate. The final study sample exceeded this required number, with 888 students completing the survey.

### 2.3. Data Collection Procedure and Collected Measures

Data were collected using paper-and-pencil questionnaires administered in a controlled classroom setting. This approach was selected on methodological grounds, as in-person classroom administration yields substantially higher response rates than web-based alternatives [31] and ensures uniformity of conditions across participants, thereby minimizing the environmental variability that can introduce systematic error into self-report data.

Surveys were distributed in lecture halls and on university premises across two academic years, with prior permission from the relevant course instructors and faculty administrators.

To minimize response biases inherent to in-person testing, a structured administration protocol was followed throughout. Research staff personally distributed the questionnaire booklets and ensured sufficient physical spacing between students to protect privacy during completion. Prior to survey administration, all participants were informed that participation was entirely voluntary and that strict anonymity and confidentiality would be maintained; no identifying information—such as names or student ID numbers—was collected at any point. Informed consent was operationalized as the act of submitting a completed questionnaire. Upon completion, participants placed their questionnaires directly into a sealed ballot box to further safeguard anonymity.

The study was conducted in accordance with the Declaration of Helsinki and approved by the Ethics Committee of the University of Split School of Medicine (No: 2181-198-03-04-22-0005) on 15 June 2021.

#### 2.3.1. The Questionnaire Assessed Several Demographic and Background Characteristics, Including Age, Gender, Year of Study, Upbringing in a Two-Parent Household, Exposure to Traumatic Events, and Prior Use of Psychological Help

Trauma exposure was assessed using a two-steps procedure. First, participants indicated whether they had ever experienced a traumatic event using a dichotomous yes/no item. Participants who responded affirmatively were then asked to specify the reason or type of trauma in an open-ended response and could report more than one event. All responses were reviewed and coded by a psychiatrist into the following categories: loss of a loved one, abuse or bullying, parental separation or divorce involving alienation, traffic accident, traumatic relocation, illness, conflict with the law, and other or insufficiently specified trauma. For the sensitivity analysis, an additional binary indicator of potentially complex trauma was created and coded as present when participants reported abuse or bullying, parental separation or divorce involving alienation, or chronic illness. Because this classification was derived from brief open-ended descriptions rather than from a validated instrument specifically assessing developmental or complex trauma, it was treated as an exploratory.

In addition to these items, the assessment battery included two standardized psychological instruments: the Pathological Narcissism Inventory (PNI) and the Brief Symptom Inventory (BSI).

#### 2.3.2. PNI (Pathological Narcissism Inventory)

The PNI is a 52-item self-report instrument designed to assess narcissistic traits across two higher-order dimensions: grandiose narcissism and vulnerable narcissism. All items are rated on a 6-point Likert scale ranging from 0 (not at all like me) to 5 (very much like me). The instrument captures seven facets of pathological narcissism: four associated with grandiosity—Entitlement Rage, Exploitativeness, Grandiose Fantasy, and Self-Sacrificing Self-Enhancement—and three associated with vulnerability—Contingent Self-Esteem, Hiding the Self, and Devaluing. Whereas the Narcissistic Personality Inventory (NPI) has traditionally been used to measure narcissism as a personality trait in general population samples, the PNI was developed specifically to capture clinically relevant, pathological features of narcissism, which was the goal of the present study.

The PNI is psychometrically robust, widely used in both clinical and research contexts, and validated across multiple languages and populations, offering comprehensive coverage of multifaceted narcissistic pathology. The instrument has been extensively used and validated in university settings to examine narcissistic traits and their associations with a range of psychological and behavioral outcomes [32,33,34,35,36,37,38]. The present study employed the validated Croatian adaptation of the PNI, which has previously been validated in university student populations [32].

#### 2.3.3. BSI (Brief Symptom Inventory)

The BSI is a widely used self-report measure of psychological distress and symptoms of psychopathology [39,40,41]. Derived as an abbreviated version of the Symptom Checklist-90, it consists of 53 items and typically requires 8–12 min to complete. The BSI may be administered either as a self-report measure or through a clinician-administered interview. It assesses emotional and behavioral functioning across nine symptom dimensions: Somatization, Obsessive-Compulsion, Interpersonal Sensitivity, Depression, Anxiety, Hostility, Phobic Anxiety, Paranoid Ideation, and Psychoticism. The instrument is suitable for both clinical and non-clinical populations, including medical patients and research participants. Respondents rate each item on a 5-point Likert scale ranging from 0 (not at all) to 4 (extremely). In accordance with the BSI manual, a T-score of 63 or above is the operationally defined cutoff for clinically significant psychological distress [39].

### 2.4. Data Transformation and Statistical Analysis

Statistical analyses were conducted using IBM SPSS Statistics, version 29 (IBM Corp., Armonk, NY, USA).

Descriptive statistics were used to summarize participant characteristics. Categorical variables are presented as frequencies and percentages, while continuous variables are reported as means and standard deviations (M ± SD) for normally distributed data or as medians and interquartile ranges (Mdn, IQR) for non-normally distributed data.

The difference between grandiose and vulnerable narcissism scores was examined using a paired *t*-test. Associations between overall pathological narcissism, narcissistic dimensions, and psychological distress were assessed using Pearson’s correlation coefficients.

To identify factors strongly associated with pathological narcissism, multivariable general linear regression models were constructed with total pathological narcissism (PNI Total), grandiose narcissism, and vulnerable narcissism as dependent variables. Candidate corelates included sociodemographic characteristics (age, sex, faculty, type of secondary school, relationship status), family characteristics (family integrity, perceived family relationship quality, number of siblings), and health-related variables (trauma exposure, previous psychological help, insomnia, and chronic fatigue). Categorical variables were entered as dummy variables using appropriate reference categories.

To examine factors associated with psychological distress, hierarchical multivariable linear regression analysis was performed with the Brief Symptom Inventory Global Severity Index (BSI-GSI) as the dependent variable. Demographic and context variables were entered first (Block 1), followed by health- and trauma-related variables (Block 2), family relationship quality (Block 3), and finally total pathological narcissism (PNI Total) in the final step to determine its incremental contribution to the explained variance. Additional models were performed using grandiose and vulnerable narcissism separately in place of the total PNI score.

Multicollinearity of these models was assessed using tolerance and variance inflation factors. Model fit was evaluated using the coefficient of determination (R^2^).

Statistical significance was set at *p* < 0.05.

## 3. Results

### 3.1. Sample Description

As shown in Table 1 the sample comprised 888 university students with a mean age of 21.3 years (SD = 2.22). The majority of participants were female (78.5%). Nearly half of the students were enrolled at the Faculty of Humanities and Social Sciences, followed by the School of Medicine and the Faculty of Civil Engineering, Architecture, and Geodesy. First- and third-year students were the most represented in the sample.

Most participants reported growing up in intact families (92%) and described their family relationships as very good or good (87%). Students most commonly discussed personal problems with friends (59%) and mothers (55%), while very few turned to teachers or faculty (0.7%). The median household size was 4 members (IQR = 4–5), with a median of one sibling (IQR = 1–2).

Regarding health-related characteristics, nearly half of the sample reported prior trauma exposure (48%), and 21% had previously sought psychological help. Of the 181 respondents who had sought psychological help, 141 (78%) reported a history of trauma. Notably, of the 426 respondents who reported trauma exposure, only 141 (33%) had sought psychological help, suggesting a substantial gap between need and help-seeking behavior.

Regarding trauma, among the 428 participants who reported experiencing trauma, the most frequently reported event was the loss of a loved one, endorsed by 183 participants (42.8% of those reporting trauma; 20.7% of the total sample), followed by abuse or bullying (154 participants or 36.0%; 16.5% of the total sample) and alienating parental separation or divorce reported (55 participants or 5.9%; 12.9% of the total sample). Most participants with a history of trauma reported one identifiable type of trauma (69.8%). Approximately 227 or 53% of all traumas were classified as complex and included reports of abuse or bullying, alienating parental separation or divorce, or chronic illness.

Psychological distress levels were moderate overall. The Global Severity Index averaged 1.19 (SD = 0.70) and the Positive Symptom Distress Index averaged 1.95 (SD = 0.53), indicating that symptoms were sufficiently pronounced to potentially affect academic functioning, interpersonal relationships, and daily activities, and may warrant professional support. Descriptive analysis revealed that 54% of participants met the criteria for elevated psychopathology. Anxiety and somatization showed the highest subscale means, while phobic anxiety was the lowest.

Pathological narcissism scores were moderate overall (PNI Total: M = 2.13, SD = 0.82). Vulnerable narcissism scores were significantly higher than grandiose narcissism scores (M = 2.40, SD = 0.80 vs. M = 1.99, SD = 0.87), with a mean difference of 0.41 (95% CI: 0.38–0.43, *p* < 0.001). Notably, the correlation between the two dimensions was exceptionally strong (r = 0.884, *p* < 0.001).

### 3.2. Correlates of Pathological Narcissism

A multivariable general linear model was conducted to examine sociodemographic, familial, and health-related correlates of pathological narcissism (PNI Total; Table 2).

Several variables emerged as significant correlates. Faculty affiliation was associated with narcissism levels: students from the Faculty of Humanities and Social Sciences reported significantly higher scores than those from the School of Medicine, whereas Civil Engineering students did not differ significantly from the reference group. Age was a modest but statistically significant negative correlate, with older participants reporting slightly lower narcissism scores. Both variables, however, accounted for only a modest proportion of variance (ηp^2^ from 0.008 to 0.019).

The family relational context was also modestly associated with pathological narcissism. Worse perceived family relationship quality was associated with higher narcissism scores. Additionally, participants who did not report trauma exposure had significantly lower narcissism scores than those who were exposed. Interestingly, when the binary indicator of any trauma exposure was replaced with a binary indicator of complex trauma exposure in the full-sample model, the model based on any trauma exposure showed slightly better performance. It explained 15.2% of the variance, compared with 14.8% for the complex-trauma model, and the trauma predictor showed a slightly larger effect (ηp^2^ = 0.009 vs. 0.007; B = −0.161 vs. −0.149) and stronger statistical significance (*p* = 0.006 vs. 0.020).

Health-related variables showed associations with pathological narcissism traits ranging from modest to moderate in magnitude. Prior psychological help-seeking was significantly associated with narcissism, with students who had not sought help reporting lower narcissism scores than those who had. The absence of insomnia was similarly associated with lower narcissism scores, while the absence of chronic fatigue demonstrated the strongest association in the model, B = −0.321, SE = 0.059, *p* < 0.001, ηp^2^ = 0.036, although the magnitude remained small.

The overall model accounted for 14% of the variance in pathological narcissism (PNI Total), reflecting a moderate level of explanatory power. Taken together, the included sociodemographic, familial, and health-related variables explained a meaningful, though not exhaustive, proportion of individual differences in narcissistic pathology.

Gender, secondary school type (gymnasium vs. vocational), relationship status, family intactness, and number of siblings were all non-significant predictors (all *p* ≥ 0.217).

Models for the grandiose and vulnerable narcissism dimensions (Supplementary Tables S1 and S2) performed similarly to the PNI Total model, retaining the same set of significant correlates with comparable effect sizes and only minor differences—most notably, age was not a significant correlate of vulnerable narcissism. All three general linear models were statistically significant: the grandiose narcissism model explained the largest proportion of variance (R^2^ = 0.162), followed by total narcissism (R^2^ = 0.152) and vulnerable narcissism (R^2^ = 0.141). The pattern of significant correlates was largely consistent across all three models, with chronic fatigue, trauma exposure, perceived family relationship quality, prior psychological help-seeking, insomnia, and faculty affiliation emerging as stable predictors. Chronic fatigue demonstrated the strongest individual contribution across all three models, while sociodemographic variables were generally non-significant, with the exception of age in the grandiose and total narcissism models.

To examine whether these associations were uniform across the lower-order factors of pathological narcissism or specific to certain components, bivariate correlations between the sociodemographic and clinical variables and the seven grandiosity and vulnerability subscales were additionally examined (Supplementary Table S3). Overall, the pattern of correlates converged with the regression findings: faculty affiliation, trauma exposure, family relationship quality, chronic fatigue, and insomnia were consistently associated with nearly all facets, mirroring their role as stable predictors of PNI Total. However, some divergences emerged. Age, which was a significant negative predictor of PNI Total in the multivariable model, showed negligible and non-significant correlations with all lower-order facets, suggesting that its association with narcissism only emerges once other correlated variables are held constant, rather than reflecting a robust direct relationship with any specific factor. Gender showed the opposite pattern: although non-significant in the multivariable model, it was weakly but significantly correlated with several facets (e.g., Exploitativeness, Contingent Self-Esteem, Devaluing), indicating that its raw association with narcissism is likely accounted for by its overlap with other predictors rather than representing an independent effect. Psychological help-seeking, while significant in both analyses, was more consistently and strongly related to PNI Total than to individual facets, where its association reached significance for only a subset (Exploitativeness, Grandiose Fantasy, Hiding the Self, Entitlement Rage), consistent with a modest effect distributed across facets that becomes more detectable at the level of the aggregate score. A sex-stratified analysis is also presented in Supplementary (Supplementary Tables S5 and S6, and paragraph Associations of Narcissism Dimensions With Psychological Distress: A Sex-Stratified Analysis).

### 3.3. Intercorrelation of Narcissism Dimensions and Their Associations with Psychological Distress

The correlation between grandiose and vulnerable narcissism scores was strong (r = 0.884, *p* < 0.001). Consistent with this, 168 participants (20%) scored high on both dimensions simultaneously.

**Figure 1:**
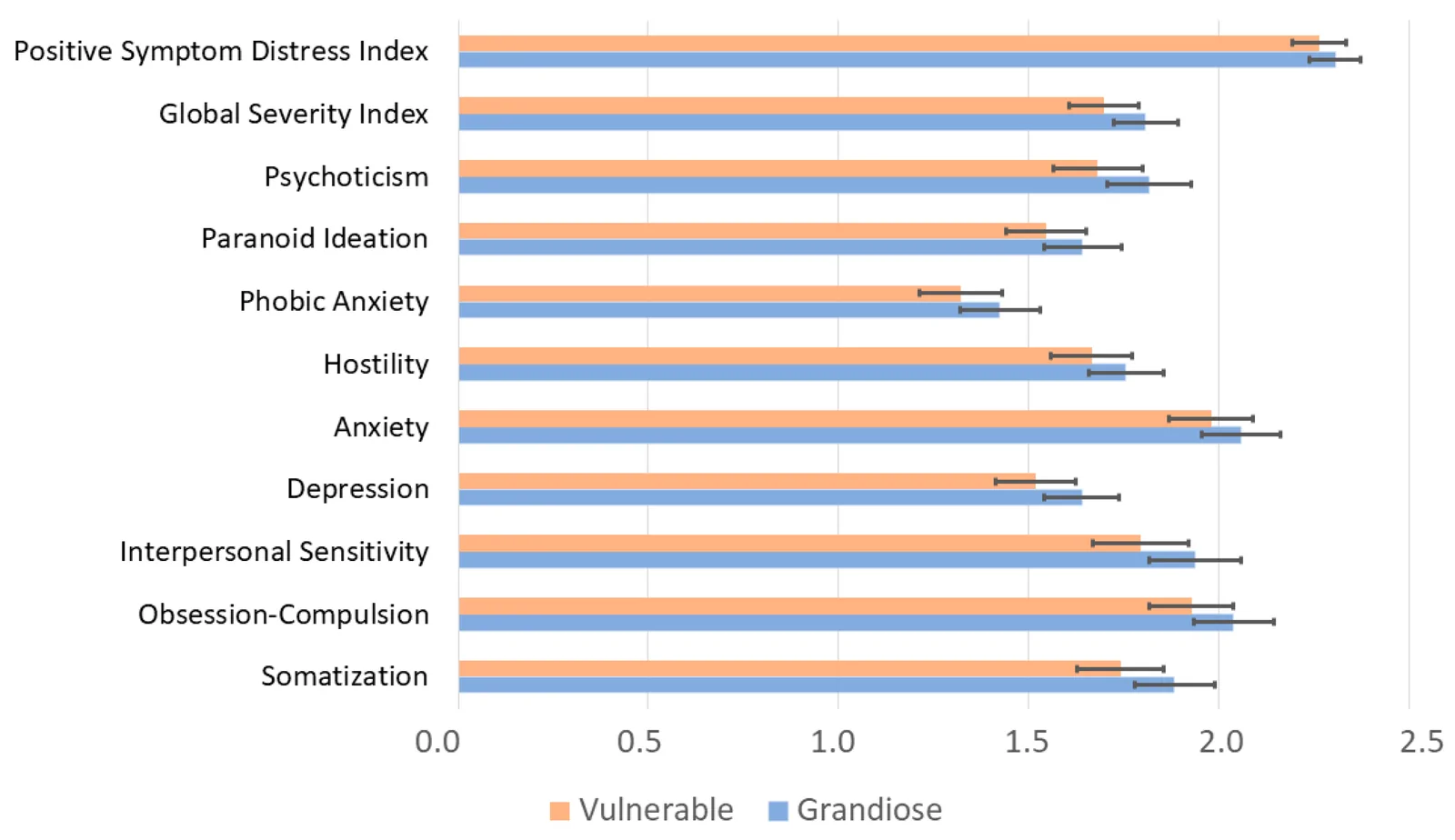
BSI scores for people with high grandiose (blue) and high vulnerable (orange) traits.

The analysis was then extended to examine the associations of grandiose, vulnerable, and overall pathological narcissism with BSI scores. Correlational analyses revealed a consistent pattern in which narcissistic personality features showed stronger associations with global psychological distress than with individual symptom domains. Grandiose narcissism demonstrated moderate to strong correlations with BSI subscales (r = 0.479–0.617) and a robust association with the Global Severity Index (r = 0.651). Vulnerable narcissism was similarly associated with all BSI dimensions (r = 0.447–0.556) and with the Global Severity Index (r = 0.581), though effect sizes were somewhat smaller than those observed for grandiose narcissism. The PNI total score showed strong positive correlations across all BSI symptom domains (r = 0.471–0.604) and with the Global Severity Index (r = 0.636), reflecting the combined contribution of grandiose and vulnerable features. In contrast, correlations at the level of individual BSI subscales were more modest (r = 0.229–0.366), though remained statistically significant across all dimensions (Supplementary Table S4). This pattern is consistent with the psychometric expectation that global indices, by aggregating information across symptom domains and items, yield higher reliability and a broader variance coverage, thereby providing a more sensitive index of association with broad personality constructs such as narcissism. Taken together, these findings suggest that narcissistic features—whether grandiose, vulnerable, or as captured by a composite score—are more strongly linked to overall psychological symptom burden than to any specific symptom cluster, pointing to a transdiagnostic rather than symptom-specific relationship between narcissism and psychopathology. Specifically, higher levels of pathological narcissism were associated with elevated psychological symptomatology across somatic, affective, interpersonal, and psychotic-like domains, with somewhat stronger associations observed for grandiose traits in this sample. Mean BSI scores with 95% confidence intervals for participants with high grandiose and high vulnerable narcissism (top 25%) are presented in Figure 1.

### 3.4. Correlates of Psychological Distress: Comparison Across PNI Models

A hierarchical multivariable regression analysis was conducted to examine demographic, health-related, relational, and personality correlates of global psychological distress (BSI–GSI). We compared three models, each using a different measure of pathological narcissism as the predictor: PNI Total, PNI Grandiosity, and PNI Vulnerability, while controlling for demographic, health-related, trauma, and family relationship variables. The model using PNI Grandiosity showed the best fit, explaining 56.2% of the variance in distress (F(12, 821) = 87.778, *p* < 0.001; Table 3). In this model, PNI Grandiosity was the strongest correlate of distress (B = 0.420, β = 0.524, *p* < 0.001), accounting for 23.0% of the explained variance on its own (ΔR^2^).

Higher distress was also independently associated with chronic fatigue, poorer family relationship quality, prior psychological help-seeking, insomnia, and trauma exposure. Among demographic and context variables, faculty affiliation, household size, gender and relationship status were statistically significant, whereas age was not. Among all these covariates, chronic fatigue contributed the most explained variance (ΔR^2^ = 3.2%), followed by family relationship quality (1.3%) and faculty affiliation (0.9–1.2%); all remaining variables contributed less.

Across the three models in which PNI Total (Supplementary Table S7), Grandiosity (Table 3), and Vulnerability (Supplementary Table S8) scores were each entered as the primary predictor, the proportion of explained variance was largely comparable. The model incorporating grandiose narcissism demonstrated the highest explanatory power (R^2^ = 56%), followed by the model using the PNI total score (55%), while the model incorporating vulnerable narcissism yielded the lowest R^2^ (51%). These differences reflected only minor variation in the overall model fit (see Supplementary Table S9 for integrated comparison; individual model results can be found in Table 3 and Supplementary Tables S7 and S8). The same set of covariates was retained across all three models, and parameter estimates remained substantively consistent. However, the incremental variance (ΔR^2^) explained by the addition of the PNI predictor varied somewhat more across models: 17.9% for vulnerability, 21.9% for total, and 23.0% for grandiosity.

## 4. Discussion

### 4.1. The Fragility of the Narcissistic Self: Linking PNI and BSI

A primary finding of this study is the robust positive correlation between pathological narcissism and global psychological distress across somatic, affective, interpersonal, and psychotic-like symptom domains, with somewhat stronger associations observed for grandiose traits in this sample. The magnitude of this association (r = 0.636) challenges the traditional view that narcissism—particularly in its grandiose form—functions as a purely protective shield against distress [42]. Prior studies have typically found grandiose narcissism to be negatively associated with, or unrelated to, pathological distress [43], and the broader literature consistently predicts and demonstrates that vulnerable narcissism is a stronger predictor of psychological distress than grandiose narcissism [8].

Several features of the present study may account for this divergence. Most prior research in this area has relied on the Narcissistic Personality Inventory (NPI); the PNI grandiosity scale, by contrast, measures pathological grandiosity—a construct considerably closer to psychopathology than to adaptive functioning. Equally relevant is the nature of the outcome measure: the BSI is not solely an index of internalizing distress but also encompasses hostility, paranoid ideation, and psychoticism—domains more closely aligned with externalizing problems. Grandiose narcissism as operationalized by the PNI—through exploitativeness, devaluation, and entitlement rage—maps theoretically onto hostility and paranoid ideation more directly than does vulnerable narcissism, which is more strongly associated with depression and anxiety. It is therefore possible that grandiose narcissism is not a uniformly stronger correlate of overall distress than the existing literature would predict, but rather a stronger correlate of specific BSI domains that are externalizing in nature, with the Global Severity Index reflecting this pattern.

The sample composition and academic context offer additional explanatory leverage. With 78.5% of participants identifying as female and all operating within a high-demand academic environment, grandiose traits such as entitlement, exploitativeness, and devaluation may generate particularly intense interpersonal conflicts—with peers, professors, and colleagues—that in turn produce significant distress. Vulnerable narcissism, while chronically present, may be less situationally reactive in this context than grandiose traits that actively disrupt relationships and academic integration. Taken together, these findings suggest that while grandiosity may project a temporary facade of resilience, it constitutes a fundamentally fragile structure that remains susceptible to acute psychological suffering.

- Faculty Differences

The analysis revealed significant differences in narcissism levels based on faculty affiliation. Students enrolled at the Faculty of Humanities and Social Sciences reported significantly higher pathological narcissism scores than students at the School of Medicine (B = 0.285, *p* < 0.001, ηp^2^ = 0.020), while Civil Engineering students did not differ significantly from the medical reference group. This finding may reflect two contributing factors: first, a self-selection effect, whereby individuals with higher levels of introspective or self-focused traits are drawn to fields concerned with human identity and society; and second, differences in academic culture, whereby the rigid, hierarchical structure of medical training may suppress narcissistic expression relative to the more individualistic and critically oriented environment of the humanities [44,45]. However, given the cross-sectional nature of the data, it is not possible to determine whether faculty self-selection reflects pre-existing personality differences or whether the academic environment itself shapes these scores over time. Longitudinal designs would be needed to disentangle selection effects from socialization effects.

- Gender and Sociodemographic Variables

Gender, secondary school type, relationship status, family intactness, and number of siblings were all non-significant correlates of pathological narcissism in this sample (all *p* ≥ 0.322). The non-significance of gender is noteworthy given that the existing literature has historically reported higher narcissism—particularly grandiosity—in males. However, more recent meta-analytic evidence suggests that gender differences in narcissism are small and diminishing, particularly when vulnerable narcissism is also considered [8]. The pronounced female majority in this sample (78.5%) also substantially limits the statistical power to detect gender differences.

- Health-Related Correlates of Pathological Narcissism
- Trauma Exposure

Trauma exposure was significantly associated with higher narcissism scores across all three models—total, grandiose, and vulnerable (B = 0.162, *p* = 0.006, ηp^2^ = 0.009).

It is important to emphasize, however, that because trauma type, developmental timing, duration, and recurrence were not assessed using a validated complex-trauma instrument in our study, this classification should be considered approximate and exploratory rather than a formal measure of complex or developmental trauma. In addition, given the cross-sectional design, it remains possible that individuals with higher narcissistic traits retrospectively appraise past events as more traumatic, or that a third variable (e.g., family adversity) underlies both trauma reporting and narcissistic traits.

Although the effect size was small, this association is theoretically robust and consistent with a substantial body of evidence linking childhood and adolescent trauma to the development of narcissistic pathology, particularly its vulnerable form [27,46,47]. In the present study, trauma was assessed as a binary variable (presence/absence), which likely underestimates the true strength of the association by failing to capture the type, severity, chronicity, or developmental timing of traumatic experiences. Despite this limitation, the direction of the association aligns with theoretical models positing that trauma disrupts self-regulatory development and may contribute to the oscillation between grandiose defenses and vulnerable self-states that characterizes pathological narcissism [48]. However, given the cross-sectional design, it remains entirely possible that individuals with higher narcissistic traits retrospectively appraise past experiences as more traumatic, or that a third variable—such as family adversity—underlies both.

- Insomnia and Chronic Fatigue

Both insomnia (B = −0.204, *p* = 0.016, ηp^2^ = 0.007) and chronic fatigue (B = −0.319, *p* < 0.001, ηp^2^ = 0.035) were associated with higher narcissism scores in the multivariable model, with chronic fatigue demonstrating the strongest individual contribution of any predictor across all three narcissism models. This pattern of association is theoretically consistent with several mechanisms. Narcissistic pathology—particularly the vulnerable dimension—is characterized by chronic emotional dysregulation, hypervigilance to social evaluation, and ruminative cognitive processing [49], all of which are known to disrupt sleep architecture and contribute to fatigue. Conversely, sleep deprivation and fatigue impair cognitive and emotional self-regulation [50], potentially exacerbating the self-esteem instability and reactive distress characteristic of narcissistic vulnerability [51].

The role of chronic fatigue as a significant correlate of both narcissistic traits and general psychological distress points to what might be termed a “psychosomatic exhaustion cycle.” Students with elevated narcissistic traits may overextend themselves in pursuit of ego-ideals, resulting in chronic fatigue that in turn diminishes their capacity for emotional regulation. This dynamic reflects a profound interplay between personality pathology and physical exhaustion and suggests that chronic fatigue may represent a somatic signature of the sustained effort required to maintain narcissistic ego-functioning.

- Family Dynamics

The positive association between poor parental relationships and levels of pathological narcissism is consistent with previous studies. Such relationships have traditionally been associated with the development of vulnerable narcissism through mechanisms of insecure attachment [52]. This phenomenon can be interpreted through the concept of “narcissistic parental investment,” in which parents value their child primarily through the prism of achievement, creating an illusion of closeness that is nevertheless conditional [34]. At the same time, the high correlation (r = 0.88) between grandiosity and vulnerability observed in this sample suggests that this family environment functions as a fragile shield that, when it fails, gives way to significant distress [53]. On the other hand, the regression model for the BSI confirmed the classic protective role of the family, with poor relationships directly predicting greater psychological distress. This suggests that the family can serve both as a factor of emotional stability (as reflected in lower total BSI scores) and as a context for the development of pathological narcissistic traits (as reflected in higher PNI scores).

Comparing the Global Severity Index scores obtained in our sample (1.19) with those reported for student populations in Germany (0.88), Sweden (0.93), and Turkey (1.07) [54,55,56] reveals substantially higher levels of psychological distress, a finding that warrants serious attention.

The high prevalence of clinically significant psychopathology observed in this study (54%), combined with the fact that pathological narcissism and chronic fatigue account for more than half of the variance in psychological distress, carries important implications for university mental health policy.

First, the exceptionally strong correlation between grandiose and vulnerable narcissism suggests that professionals working in Student Counseling Centers should not confine their attention to the stereotypically grandiose presentation; rather, they should remain alert to the possibility that an outward mask of self-confidence may conceal profound emotional fragility that can readily escalate into clinical distress. Second, given that chronic fatigue emerged as a strong predictor of both narcissism and distress, prevention programs should integrate stress management interventions and sleep hygiene education alongside psychological support.

Particularly concerning is the finding that students place little trust in their professors and rarely—in fewer than 1% of cases—turn to them for help, even while reporting somatic symptoms and persistent fatigue. This underscores the need for active destigmatization of help-seeking behavior at the university level. Rather than focusing exclusively on academic performance, higher education institutions should implement screening tools capable of identifying, at an early stage, the combination of psychological vulnerability and physical exhaustion. Such an integrated approach—one that accounts for the interplay among narcissistic dynamics, family relationships, and physical health—is essential for the effective prevention of serious psychopathological outcomes in the student population.

### 4.2. Limitation

Notwithstanding the strengths of this study—including a large community-based sample, strong explanatory power, and the simultaneous examination of multiple narcissism dimensions—several limitations must be considered when interpreting the findings.

First, the cross-sectional design precludes any causal inferences regarding the relationships between pathological narcissism, psychological distress, and somatic symptoms. Although the multivariable models identified chronic fatigue and insomnia as significant correlates of narcissistic traits, the cross-sectional design of this study precludes any inference regarding the directionality of these associations. It remains equally plausible that somatic complaints contribute to narcissistic vulnerability, that psychological strain associated with narcissistic pathology manifests somatically, or that both reflect shared underlying mechanisms. Longitudinal research is needed to clarify the nature of these relationships.

Second, all data were gathered using self-report measures, which are inherently susceptible to social desirability and recall biases. This is particularly relevant when measuring pathological narcissism and family dynamics, as individuals with high narcissistic traits may consciously or unconsciously idealize their family relationships or underreport their psychological vulnerabilities to protect their self-image. Incorporating multi-informant reports (e.g., parental or peer perspectives) or clinical interviews would strengthen future investigations. Additionally, regarding self-reporting measure, trauma exposure was not assessed with validated instrument, so classification of complex trauma classification should be considered approximate and exploratory rather than a formal measure of complex or developmental trauma.

Third, the sample is characterized by a gender imbalance, with female students comprising 78.5% of the participants. Although this reflects the typical demographic distribution across the surveyed faculties (particularly Humanities and Social Sciences), it limits the generalizability of the findings to male university students.

Fourth, the study was conducted within a specific sociocultural setting (a Mediterranean region of Croatia), where family ties are traditionally highly centralized and young adults frequently cohabitate with their parents during their studies. Because self-reported family relationship quality may be judged against different cultural reference points, the findings regarding the association between family relationship quality and narcissism may not be fully generalizable to student populations in Western cultures with higher rates of early independence and different residential patterns.

Fifth, given the broad and exploratory nature of the predictor set examined, the present findings should be regarded as hypothesis-generating rather than confirmatory. The absence of pre-specified directional hypotheses means that some associations, including those that diverge from prior literature, should be interpreted with appropriate caution and considered candidates for confirmatory replication in future, theory-driven studies rather than as established, directional effects.

### 4.3. Clinical and Educational Implications

This study contributes to the literature by providing insight into the relationship between pathological narcissistic traits, psychological distress and somatic factors within a Croatian student population.

Despite study limitations, the present findings carry several implications for university mental health services and research. The high prevalence of elevated psychopathology (54%) and the strong association of pathological narcissistic traits with global distress suggest that pathological narcissism is a clinically meaningful and under-screened dimension in student mental health assessments. Routine inclusion of a brief narcissism screening instrument—alongside standard measures of depression and anxiety—may improve the identification of students at elevated risk for persistent distress and functional impairment.

The finding that chronic fatigue and insomnia were among the most robust correlates of both narcissistic pathology and psychological distress highlights the importance of addressing somatic and sleep-related complaints within university counseling settings, as these may serve as both markers and amplifiers of underlying psychological vulnerability. Similarly, the associations with trauma exposure and poor family relationship quality suggest the potential value of trauma-informed and family-systems-oriented approaches in university mental health provision.

Future research should address the limitations of the present study by employing longitudinal designs capable of establishing temporal precedence among narcissism, distress, and their correlates. Ideally, multi-wave assessments would span the full duration of university studies to capture developmental trajectories. The inclusion of informant-rated measures, behavioral indicators, and physiological stress markers would strengthen the construct validity of key variables. Finally, replication across institutions, national contexts, and gender-balanced samples would enhance the generalizability of these findings.

## 5. Conclusions

This study highlights a critical public health challenge within higher education, revealing that 54% of university students experienced elevated levels of psychological distress, while fewer than 1% reported turning to academic staff for support when facing personal difficulties—underscoring a stark institutional disconnect that warrants systemic attention.

The findings advance the understanding of personality pathology in academic populations by demonstrating that pathological narcissistic traits represent the strongest concurrent correlate of global psychological distress, independent of health-related, trauma, and relational variables. Vulnerable narcissism exceeded grandiosity in magnitude, yet the two dimensions were highly correlated, with a notable proportion of students exhibiting both profiles simultaneously. Chronic fatigue and insomnia emerged as the strongest health-related correlates of narcissistic traits across all models, suggesting a substantial physiological burden associated with narcissistic self-structure. Sociodemographic correlates were modest by comparison, whereas trauma exposure showed more consistent associations alongside somatic complaints.

Consistent with the theoretical framing outlined in the introduction, this study focused on pathological narcissism as a dimensional personality trait rather than as a categorical clinical diagnosis of narcissistic personality disorder. Accordingly, the findings should not be interpreted as suggesting that narcissistic traits are inherently pathological. Healthy self-esteem and adaptive self-confidence lie along the same broad trait continuum, yet remain distinct from its pathological expression, which is characterized by rigidity, interpersonal exploitation, and contingent self-worth. It was specifically this dysregulated, pathological form—rather than self-confidence or ambition per se—that was associated with elevated psychological distress and somatic burden in the present sample. Consequently, it is this pathological expression of narcissism, rather than the trait in its broader, non-pathological sense that constitutes the primary target of the screening and intervention strategies discussed below.

From a public health perspective, these findings underscore the need for university healthcare systems to move beyond passive intervention models. Campus counseling and medical centers should consider implementing proactive, integrative screening protocols that recognize chronic somatic complaints—such as fatigue and sleep disturbances—as potential markers of underlying psychological distress and personality vulnerabilities. Tailored interventions addressing both somatic and personality-level needs are warranted to foster resilience and improve student well-being. In the absence of intervention studies specifically targeting pathological narcissism as a personality trait in university populations (as opposed to the existing clinical literature suggesting approaches such as mentalization, schema therapy, and transference-focused psychotherapy) at the preventive, nonclinical level, university programs that build emotional regulation skills, realistic self-appraisals, and constructive responses to failure or criticism—rather than programs aimed at reducing self-esteem or ambition per se—may convincingly reduce the escalation of vulnerable narcissistic traits into clinically significant distress. Controlled evaluation of such preventive programs within a university counseling setting represents an important direction for future research.

## Figures and Tables

**Table 1:** Sample Characteristics (N = 888).

		Statistics
Sociodemographic Characteristics		
Age (years), M ± SD		21.3 (2.22)
Gender, N (%)		
Female	697	78.5
Male	190	21.4
Faculty/School, N (%)		
Faculty of Humanities and Social Sciences	400	45.1
School of Medicine	262	29.5
Faculty of Civil Engineering	226	25.5
Year of study, N (%)		
1st	335	37.7
2nd	49	5.5
3rd	269	30.3
4th	73	8.2
5th	95	10.7
6th	67	7.6
Secondary school type, N (%)		
Grammar school (Gymnasium)	642	72.5
Applied/Vocational school	243	27.5
Relationship status, N (%)		
In a relationship	515	58.3
Single	355	40.2
Married	10	1.1
Other	3	0.3
Family Context		
Intact family *, N (%)	818	92.1
Perceived family relationship quality, N (%)		
Very good	498	56.3
Good	270	30.5
Average	91	10.3
Poor	23	2.6
Very poor	3	0.3
Discusses personal problems with, N (%) **		
Mother	486	54.7
Friend	526	59.2
Sibling	258	29.1
Father	146	16.5
Other	127	14.3
Teacher/professor	6	0.7
Household size, Median (IQR)		4 (4–5)
Number of siblings, Median (IQR)		1 (1–2)
Health and Psychological Status		
Insomnia, N (%)	119	13.4
Chronic fatigue, N (%)	379	42.7
Trauma exposure, N (%)	428	48.2
Complex trauma **		
Abuse/bullying	154	17.4
Alienating parental separation or divorce	55	6.2
Chronic illness	25	2.8
Other trauma **		0.0
Loss of a loved one	183	20.7
Traffic accident	44	5.0
Traumatic relocation	32	3.6
Conflict with the law	4	0.5
Type of trauma not clearly specified	53	6.0
Prior psychological help-seeking, N (%)	183	20.6
Psychological Distress (BSI)		
Elevated psychopathology (BSI cutoff), N (%)	477	53.7
Global Severity Index (GSI), M ± SD		1.19 (0.70)
Positive Symptom Distress Index (PSDI), M ± SD		1.95 (0.53)
Somatization, M ± SD		1.22 (0.83)
Obsessive-Compulsive, Median (IQR)		1.33 (0.83–2.00)
Interpersonal Sensitivity, M ± SD		1.20 (0.93)
Depression, M ± SD		1.06 (0.75)
Anxiety, M ± SD		1.35 (0.85)
Hostility, M ± SD		1.18 (0.79)
Phobic Anxiety, M ± SD		0.91 (0.75)
Paranoid Ideation, M ± SD		1.05 (0.76)
Psychoticism, M ± SD		1.20 (0.85)
Pathological Narcissism (PNI)		
Contingent Self-Esteem (CSE), M ± SD		2.71 (0.88)
Exploitativeness (EXP), M ± SD		2.21 (1.08)
Self-Sacrificing Self-Enhancement (SSSE), M ± SD		1.85 (1.03)
Hiding the Self (HS), M ± SD		2.24 (0.83)
Grandiose Fantasy (GF), M ± SD		2.05 (0.82)
Devaluing (DEV), M ± SD		2.24 (0.99)
Entitlement Rage (ER), M ± SD		1.86 (1.06)
Grandiosity composite, M ± SD		1.99 (0.87)
Vulnerability composite, M ± SD		2.40 (0.80)
PNI Total, M ± SD		2.13 (0.82)

Note. BSI = Brief Symptom Inventory; PNI = Pathological Narcissism Inventory; M = arithmetic mean; SD = standard deviation; IQR = interquartile range. Percentages are based on valid cases; missing data ranged from 0–3%, and may not sum to exactly 100% due to rounding. * Intact family was defined as parents living together at the time of the study. ** Multiple responses were permitted; thus percentages sum to more than 100%.

**Table 2:** Factors Associated with Pathological Narcissism.

Factor	B	SE	t	*p*	95% CI	ηp^2^
Faculty						
Faculty (HSS vs. MF)	0.280	0.071	3.959	<0.001	[0.141, 0.418]	0.019
Faculty (CE vs. MF)	−0.047	0.077	−0.607	0.544	[−0.198, 0.105]	0.000
Gender (Females vs. Males)	−0.011	0.070	−0.164	0.870	[−0.148, 0.125]	0.000
High School (Applied/Vocational vs. Gymnasium)	−0.037	0.065	−0.573	0.567	[−0.164, 0.09]	0.000
Intact family (No vs. Yes)	−0.055	0.106	−0.521	0.603	[−0.263, 0.153]	0.000
Trauma (No vs. Yes)	−0.161	0.059	−2.751	0.006	[−0.276, −0.046]	0.009
Relationship status						
(Single vs. in a relationship)	−0.068	0.055	−1.236	0.217	[−0.176, 0.04]	0.002
Family relationship quality						
(higher score = poorer relationship)	0.092	0.037	2.496	0.013	[0.02, 0.163]	0.008
Psychological help-seeking (No vs. Yes)	−0.208	0.070	−2.956	0.003	[−0.346, −0.07]	0.011
Number of siblings	−0.024	0.022	−1.086	0.278	[−0.068, 0.019]	0.001
Insomnia (No vs. Yes)	−0.203	0.084	−2.407	0.016	[−0.369, −0.038]	0.007
Chronic fatigue (No vs. Yes)	−0.321	0.059	−5.489	<0.001	[−0.436, −0.207]	0.036
Age	−0.031	0.012	−2.488	0.013	[−0.055, −0.006]	0.008

Note. Dependent variable: PNI Total score (mean across N1–N52). Dummy-coded predictors are compared to their respective reference categories. HSS = Humanities and Social Sciences; MF = School of Medicine; CE = Civil Engineering; B = unstandardized coefficient; SE = standard error; β = standardized coefficient; CI = confidence intervals; ηp^2^ = partial eta squared.

**Table 3:** Factors Associated with Psychological Distress (BSI–GSI): Model Including PNI Grandiosity.

Variables	B	SE	β	t	*p*	95% CI for B	ΔR^2^	Block
Demographic/Context Variables								
Age	−0.001	0.007	−0.003	−0.107	0.915	[−0.015, 0.014]	0.000	1
Gender (Male vs. Female)	−0.127	0.042	−0.074	−3.020	0.003	[−0.210, −0.045]	0.005	1
Faculty (HSS vs. FM)	0.167	0.040	0.120	4.157	<0.001	[0.088, 0.246]	0.009	1
Faculty (CE vs. FM)	0.211	0.045	0.129	4.698	<0.001	[0.123, 0.300]	0.012	1
Relationship status (Single vs. In a relationship)	−0.084	0.034	−0.059	−2.512	0.012	[−0.150, −0.018]	0.003	1
Number of Family Members	0.039	0.012	0.078	3.322	<0.001	[0.016, 0.062]	0.006	1
Health/Trauma Variables								
Psychological help-seeking (Yes vs. No)	0.132	0.043	0.076	3.056	0.002	[0.047, 0.216]	0.005	2
Trauma exposure (Yes vs. No)	0.089	0.036	0.064	2.480	0.013	[0.019, 0.159]	0.003	2
Insomnia (Yes vs. No)	0.153	0.051	0.074	2.992	0.003	[0.052, 0.253]	0.005	2
Chronic Fatigue (Yes vs. No)	0.282	0.036	0.200	7.813	<0.001	[0.211, 0.353]	0.032	2
Relationship Functioning								
Family relationship quality (higher score = poorer relationship)	0.109	0.022	0.123	5.031	<0.001	[0.067, 0.152]	0.013	3
Personality								
PNI Grandiosity (mean of subscales)	0.420	0.020	0.524	20.769	<0.001	[0.380, 0.460]	0.230	4
Model summary R^2^ = 0.562; Adjusted R^2^ = 0.556; ΔR^2^ (Block 4) = 0.230; F change = 431,336 (df1 = 1, df2 = 821); *p* < 0.001 Block 1: F change (6; 827) = 12.35 *p* < 0.001 Block 2: F change (10; 823) = 36.531 *p* < 0.001 Block 3: F change (11; 822) = 37.115 *p* < 0.001 Block 4: F change(12; 821) = 87.778 *p* < 0.001 Multicollinearity diagnostics were acceptable (Tolerance = 0.647–.965; VIF = 1.036–1.546), all VIFs ≤ 1.23.								

Note. B = unstandardized coefficient; SE = standard error; β = standardized coefficient.

## Data Availability

The datasets generated and analyzed during the current study are not publicly available due to privacy and ethical restrictions but are available from the corresponding author on reasonable request.

## Supplementary Materials

The following supporting information can be downloaded at: https://www.mdpi.com/article/10.3390/healthcare14162470/s1, Supplementary Table S1. Factors Associated with Pathological Narcissism—Grandiosity dimension. Supplementary Table S2. Factors Associated with Pathological Narcissism—Vulnerability dimension. Supplementary Table S3. Associations of Sociodemographic and Clinical Characteristics with Pathological Narcissism Inventory Lower-Order Factors in the Full Sample. Supplementary Table S4. Correlations Between Brief Symptom Inventory and Pathological Narcissism Inventory Scores—Lower Order Factors. Supplementary Table S5. Correlations Between Brief Symptom Inventory and Pathological Narcissism Inventory Scores Among Women—Lower Order Factors. Supplementary Table S6. Correlations Between Brief Symptom Inventory Scores and Pathological Narcissism Inventory Lower-Order Factors Among Men. Supplementary Table S7. Factors Associated with Psychological Distress: PNI Total Model. Supplementary Table S8. Factors Associated with Psychological Distress: PNI Vulnerability Model. Supplementary Table S9. Summary Table—Comparison of Regression Models Predicting Psychological Distress (BSI-GSI) Across PNI Operationalizations.

## Abbreviations

The following abbreviations are used in this manuscript:

PNI	Pathological Narcissism Inventory
BSI	Brief Symptom Inventory
