# Supplementary material for: Pathological Narcissism and Psychological Distress Among University Students: The Interplay of Family Dynamics, Trauma, and Health-Related Factors
Source: Healthcare (Basel). 2026 Aug 10;14(16):2470. doi: 10.3390/healthcare14162470 (PMC13512590; doi:10.3390/healthcare14162470)
Supplement: Supplementary file 1 [file healthcare-14-02470-s001.zip › healthcare-4437269-supplementary.pdf]

**Supplementary material, Krnić et al. (2026)**

**Associations of Narcissism Dimensions with Psychological Distress: a Sex-Stratified Analysis**

Across the full sample (Supplementary Table S4.) and among women (Supplementary Table S5.), all correlations between BSI scores and PNI lower-order factors were positive and generally small to moderate (full sample: correlations ranging from $r$ = .229 to .366 and averaging approximately $r$ = .31., women: correlations ranging from $r$ = .235 to .393 and averaging r = .32), with the strongest associations involving the Global Severity Index and Grandiose Fantasy.

The male subsample (Supplementary Table S6.) showed a similar overall pattern, although several associations involving Phobic Anxiety did not reach the conventional $p < .05$ threshold. However, these differences in statistical significance should not be interpreted as evidence of true sex differences, as the smaller male sample of N=190 was underpowered to detect correlation coefficients below r = .20. Namely, the non-significant estimates in the male subsample (r = .124–.173) fall just below the critical value for this sample size, consistent with a power limitation rather than a genuine absence of association.

**TABLES:**

**Supplementary Table S1. Factors Associated with Pathological Narcissism – Grandiosity dimension**

| Factor | *B* | *SE* | *t* | *p* | *95% CI* | $\eta p^2$ |
|---|---|---|---|---|---|---|
| Faculty | | | | | | |
| Faculty (HSS vs MF) | 0,325 | 0,074 | 4,391 | <0,001 | [0,18, 0,471] | 0,023 |
| Faculty (CE vs MF) | -0,004 | 0,081 | -0,050 | 0,960 | [-0,163, 0,155] | 0,000 |
| Gender (Females vs Males) | -0,028 | 0,073 | -0,382 | 0,702 | [-0,172, 0,116] | 0,000 |
| High School | | | | | | |
| (Applied/Vocational vs Gymnasium) | -0,033 | 0,068 | -0,485 | 0,628 | [-0,166, 0,1] | 0,000 |
| Intact family (No vs Yes) | -0,061 | 0,111 | -0,545 | 0,586 | [-0,279, 0,158] | 0,000 |
| Trauma (No vs Yes) | -0,188 | 0,061 | -3,065 | 0,002 | [-0,309, -0,068] | 0,011 |
| Relationship status | | | | | | |

| | | | | | | |
|---|---|---|---|---|---|---|
| (Single vs In a relationship) | -0,074 | 0,058 | -1,281 | 0,201 | [-0,188, 0,039] | 0,002 |
| Family relationship quality | | | | | | |
| (higher score = poorer relationship) | 0,097 | 0,038 | 2,522 | 0,012 | [0,021, 0,172] | 0,008 |
| Psychological help-seeking (No vs Yes) | -0,248 | 0,074 | -3,360 | 0,001 | [-0,393, -0,103] | 0,014 |
| Number of siblings | -0,027 | 0,023 | -1,173 | 0,241 | [-0,073, 0,018] | 0,002 |
| Insomnia (No vs Yes) | -0,197 | 0,089 | -2,221 | 0,027 | [-0,371, -0,023] | 0,006 |
| Chronic fatigue (No vs Yes) | -0,342 | 0,061 | -5,564 | <0,001 | [-0,463, -0,221] | 0,037 |
| Age | -0,037 | 0,013 | -2,878 | 0,004 | [-0,063, -0,012] | 0,010 |

**Note.** Dependent variable: PNI Total score. Dummy-coded predictors are compared to their respective reference categories. HSS = Humanities and Social Sciences; MF = School of Medicine; CE = Civil Engineering; CI = confidence intervals; $\eta p^2$ = partial eta squared.

**Supplementary Table S2. Factors Associated with Pathological Narcissism – Vulnerability dimension**

| **Factor** | ***B*** | ***SE*** | ***t*** | ***p*** | ***95% CI*** | ***ηp²*** |
|---|---|---|---|---|---|---|
| Faculty | | | | | | |
| Faculty (HSS vs MF) | 0,268 | 0,068 | 3,916 | <0,001 | [0,134, 0,403] | 0,019 |
| Faculty (CE vs MF) | -0,082 | 0,075 | -1,100 | 0,271 | [-0,229, 0,064] | 0,001 |
| Gender (Females vs Males) | 0,062 | 0,068 | 0,917 | 0,359 | [-0,071, 0,195] | 0,001 |
| High School | | | | | | |
| (Applied/Vocational vs Gymnasium) | -0,026 | 0,063 | -0,417 | 0,677 | [-0,149, 0,097] | 0,000 |
| Intact family (No vs Yes) | -0,082 | 0,103 | -0,795 | 0,427 | [-0,283, 0,12] | 0,001 |
| Trauma (No vs Yes) | -0,136 | 0,057 | -2,399 | 0,017 | [-0,248, -0,025] | 0,007 |
| Relationship status | | | | | | |

| | | | | | | |
|---|---|---|---|---|---|---|
| (Single vs In a relationship) | -0,092 | 0,053 | -1,720 | 0,086 | [-0,197, 0,013] | 0,004 |
| Family relationship quality | | | | | | |
| (higher score = poorer relationship) | 0,072 | 0,036 | 2,039 | 0,042 | [0,003, 0,142] | 0,005 |
| Psychological help-seeking (No vs | -0,162 | 0,068 | -2,372 | 0,018 | [-0,295, -0,028] | 0,007 |
| Number of siblings | -0,004 | 0,021 | -0,170 | 0,865 | [-0,046, 0,039] | 0,000 |
| Insomnia (No vs Yes) | -0,164 | 0,082 | -2,007 | 0,045 | [-0,325, -0,004] | 0,005 |
| Chronic fatigue (No vs Yes) | -0,297 | 0,057 | -5,234 | <0,001 | [-0,409, -0,186] | 0,033 |
| Age | -0,021 | 0,012 | -1,766 | 0,078 | [-0,045, 0,002] | 0,004 |

**Supplementary Table S3. Associations of Sociodemographic and Clinical Characteristics with Pathological Narcissism Inventory Lower-Order Factors in the Full Sample**

| **Characteristic** | **Method** | **Narcissistic grandiosity** | | | **Narcissistic vulnerability** | | | |
|---|---|---|---|---|---|---|---|---|
| | | Exploitativeness | Self-Sacrificing Self-Enhancement | Grandiose Fantasy | Contingent Self-Esteem | Hiding the Self | Devaluing | Entitlement Rage |
| Faculty of Humanities and Social Sciences | *r*pb | .108** | .114** | .121** | .097** | .130** | .168** | .106** |
| Faculty of Civil Engineering | *r*pb | −.041 | −.054 | −.024 | −.032 | −.026 | −.079** | −.040 |
| Gender | *r*pb | −.080** | −.009 | −.066* | −.076** | −.066* | −.091** | −.066* |
| High School | *r*pb | −.054 | −.031 | −.045 | −.047 | −.043 | −.052 | −.053 |
| Intact family structure | *r*pb | −.023 | −.013 | −.041 | .007 | −.012 | .003 | .013 |
| History of trauma | *r*pb | .134** | .130** | .114** | .112** | .135** | .130** | .132** |
| Relationship status | *r*pb | −.029 | −.009 | −.001 | −.026 | −.024 | −.030 | −.012 |
| Relationship quality | $\rho$ | .156** | .154** | .156** | .106** | .109** | .143** | .122** |

| Characteristic | Method | Narcissistic grandiosity | | | Narcissistic vulnerability | | | |
|---|---|---|---|---|---|---|---|---|
| History of seeking psychological help | *r*pb | .075** | .035 | .074** | .037 | .065* | .047 | .064* |
| Number of siblings | *ρ* | .028 | .032 | .014 | .022 | .047 | −.023 | .022 |
| Chronic fatigue | *r*pb | .115** | .114** | .155** | .129** | .115** | .076** | .140** |
| Insomnia | *r*pb | .138** | .095** | .098** | .083** | .081** | .071** | .079** |
| Age | *ρ* | −.035 | −.052 | −.015 | −.008 | −.056* | .000 | −.001 |

*Note.* Values are point-biserial correlation coefficients (*r*pb) for dichotomous variables and Spearman rank correlation coefficients (*ρ*) for ordinal or continuous variables. ***p* < .05; **p* < .10. PNI = Pathological Narcissism Inventory. The PNI lower-order factors are grouped into the higher-order dimensions of narcissistic grandiosity and narcissistic vulnerability. The direction of point-biserial correlations depends on the coding of the dichotomous variables.

**Supplementary Table S4. Correlations Between Brief Symptom Inventory and Pathological Narcissism Inventory Scores - Lower Order Factors**

| | Pathological Narcissism Inventory Lower Order Factors | | | | | | |
|---|---|---|---|---|---|---|---|
| | Narcissistic Grandiosity | | | Narcissistic Vulnerability | | | |
| Brief Symptom Inventory score | Exploitativeness | Self-Sacrificing Self-Enhancement | Grandiose Fantasy | Contingent Self-Esteem | Hiding the Self | Devaluing | Entitlement Rage |
| Global Severity Index | 0.361 | 0.355 | 0.366 | 0.337 | 0.359 | 0.353 | 0.352 |
| Somatization | 0.326 | 0.330 | 0.338 | 0.292 | 0.335 | 0.339 | 0.332 |
| Anxiety | 0.334 | 0.327 | 0.341 | 0.329 | 0.352 | 0.340 | 0.338 |
| Depression | 0.297 | 0.298 | 0.315 | 0.281 | 0.305 | 0.295 | 0.297 |
| Interpersonal Sensitivity | 0.329 | 0.315 | 0.334 | 0.309 | 0.312 | 0.315 | 0.329 |

| | | | | | | | |
|---|---|---|---|---|---|---|---|
| Obsession–Compulsion | 0.318 | 0.315 | 0.313 | 0.292 | 0.301 | 0.318 | 0.290 |
| Psychoticism | 0.278 | 0.281 | 0.295 | 0.267 | 0.303 | 0.274 | 0.267 |
| Paranoid Ideation | 0.336 | 0.342 | 0.332 | 0.308 | 0.318 | 0.324 | 0.326 |
| Phobic Anxiety | 0.273 | 0.256 | 0.279 | 0.246 | 0.270 | 0.229 | 0.263 |
| Hostility | 0.290 | 0.289 | 0.281 | 0.283 | 0.299 | 0.269 | 0.289 |

*Note. Values are Pearson's correlation coefficients. All correlations were statistically significant at p < .001. BSI = Brief Symptom Inventory; PNI = Pathological Narcissism Inventory.*

**Supplementary Table S5. Correlations Between Brief Symptom Inventory and Pathological Narcissism Inventory Scores Among Women - Lower Order Factors**

| | Pathological Narcissism Inventory Lower Order Factors | | | | | | |
|---|---|---|---|---|---|---|---|
| | Narcissistic Grandiosity | | | Narcissistic Vulnerability | | | |
| **Brief Symptom Inventory score** | **Exploitativeness** | **Self-Sacrificing Self-Enhancement** | **Grandiose Fantasy** | **Contingent Self-Esteem** | **Hiding the Self** | **Devaluing** | **Entitlement Rage** |
| Global Severity Index | 0.363 | 0.383 | 0.393 | 0.339 | 0.361 | 0.351 | 0.357 |
| Somatization | 0.329 | 0.359 | 0.375 | 0.298 | 0.347 | 0.345 | 0.347 |
| Anxiety | 0.328 | 0.348 | 0.352 | 0.316 | 0.348 | 0.323 | 0.332 |
| Depression | 0.294 | 0.320 | 0.341 | 0.289 | 0.313 | 0.290 | 0.302 |
| Interpersonal Sensitivity | 0.318 | 0.329 | 0.342 | 0.296 | 0.307 | 0.299 | 0.314 |
| Obsession–Compulsion | 0.323 | 0.346 | 0.336 | 0.294 | 0.300 | 0.321 | 0.293 |
| Psychoticism | 0.287 | 0.309 | 0.320 | 0.285 | 0.300 | 0.277 | 0.279 |
| Paranoid Ideation | 0.332 | 0.347 | 0.348 | 0.294 | 0.313 | 0.318 | 0.321 |
| Phobic Anxiety | 0.282 | 0.286 | 0.302 | 0.259 | 0.281 | 0.235 | 0.283 |
| Hostility | 0.292 | 0.317 | 0.315 | 0.291 | 0.300 | 0.270 | 0.292 |

*Note. Values are Pearson's correlation coefficients. All correlations were statistically significant at $p < .001$. BSI = Brief Symptom Inventory; PNI = Pathological Narcissism Inventory.*

**Supplementary Table S6. Correlations Between Brief Symptom Inventory Scores and Pathological Narcissism Inventory Lower-Order Factors Among Men**

| | Pathological Narcissism Inventory Lower-Order Factors | | | | | | |
|---|---|---|---|---|---|---|---|
| | Narcissistic Grandiosity | | | Narcissistic Vulnerability | | | |
| **Brief Symptom Inventory score** | **Exploitativeness** | **Self-Sacrificing Self-Enhancement** | **Grandiose Fantasy** | **Contingent Self-Esteem** | **Hiding the Self** | **Devaluing** | **Entitlement Rage** |
| Global Severity Index | 0.314 | 0.257 | 0.226 | 0.290 | 0.318 | 0.313 | 0.302 |
| Somatization | 0.267 | 0.230 | 0.149 | 0.220 | 0.246 | 0.255 | 0.228 |
| Anxiety | 0.312 | 0.264 | 0.269 | 0.340 | 0.335 | 0.352 | 0.332 |
| Depression | 0.271 | 0.212 | 0.187 | 0.208 | 0.234 | 0.263 | 0.242 |
| Interpersonal Sensitivity | 0.331 | 0.280 | 0.275 | 0.322 | 0.297 | 0.326 | 0.365 |
| Obsession–Compulsion | 0.247 | 0.199 | 0.182 | 0.233 | 0.258 | 0.241 | 0.235 |
| Psychoticism | 0.208 | 0.167 | 0.169 | 0.163 | 0.292 | 0.224 | 0.192 |
| Paranoid Ideation | 0.332 | 0.322 | 0.258 | 0.343 | 0.315 | 0.325 | 0.333 |
| Phobic Anxiety | 0.183 | 0.129† | 0.145† | 0.140† | 0.173 | 0.135† | 0.124† |
| Hostility | 0.257 | 0.195 | 0.133† | 0.231 | 0.287 | 0.241 | 0.253 |

*Note. Values are Pearson correlation coefficients. Correlations marked with † did not reach the conventional .05 significance level but showed a marginal association at p < .10. All remaining correlations were statistically significant at p < .05. BSI = Brief Symptom Inventory; PNI = Pathological Narcissism Inventory. † p < .10.*

**Supplementary Table S7. Factors Associated With Psychological Distress: PNI Total Model**

| **Variables** | *B* | *SE* | *β* | *t* | *p* | *95% CI for B* | Δ$R^2$ | Block |
|---|---|---|---|---|---|---|---|---|
| **Demographic / Context Variables** | | | | | | | | |
| Age | -0,003 | 0,008 | -0,011 | -0,465 | 0,642 | [-0,018, 0,011] | 0,000 | 1 |
| Gender (Male vs Female) | -0,120 | 0,043 | -0,070 | -2,813 | 0,005 | [-0,204, -0,036] | 0,004 | 1 |
| Faculty (HSS vs FM) | 0,185 | 0,041 | 0,132 | 4,543 | <0,001 | [0,105, 0,265] | 0,011 | 1 |
| Faculty (CE vs FM) | 0,231 | 0,046 | 0,141 | 5,066 | <0,001 | [0,141, 0,320] | 0,014 | 1 |
| Relationship status (Single vs In a relationship) | -0,081 | 0,034 | -0,057 | -2,397 | 0,017 | [-0,148, -0,015] | 0,003 | 1 |
| Number of Family Members | 0,036 | 0,012 | 0,072 | 3,041 | 0,002 | [0,013, 0,059] | 0,005 | 1 |
| **Health / Trauma Variables** | | | | | | | | |
| Psychological help-seeking (Yes vs No) | 0,145 | 0,044 | 0,083 | 3,318 | <0,001 | [0,059, 0,230] | 0,006 | 2 |
| Trauma exposure (Yes vs No) | 0,097 | 0,036 | 0,069 | 2,680 | 0,008 | [0,026, 0,168] | 0,004 | 2 |
| Insomnia (Yes vs No) | 0,150 | 0,052 | 0,073 | 2,908 | 0,004 | [0,049, 0,252] | 0,005 | 2 |
| Chronic Fatigue (Yes vs No) | 0,287 | 0,037 | 0,203 | 7,857 | <0,001 | [0,215, 0,359] | 0,034 | 2 |
| **Relationship Functioning** | | | | | | | | |
| Family relationship quality (higher score = poorer relationship) | 0,111 | 0,022 | 0,125 | 5,054 | <0,001 | [0,068, 0,154] | 0,014 | 3 |
| **Personality** | | | | | | | | |
| PNI Total (mean across N1-N52) | 0,430 | 0,021 | 0,508 | 20,022 | <0,001 | [0,388, 0,472] | 0,219 | 4 |

**Model summary**

$R^2$ = 0,551; Adjusted $R^2$ = 0,544; Δ$R^2$ (Block 4) = 0,219; F change = 400,871 (df1=1, df2=821); p < 0,001

*Block 1: F change (6; 827) = 12,35 p<,001*

*Block 2: F change (10; 823) = 36,531 p<,001*

*Block 3: F change (11; 822) = 37,115 p<,001*

*Block 4: F change (12; 821) = 83,978 p<,001*

| Variables | *B* | *SE* | *β* | *t* | *p* | *95% CI for B* | $\Delta R^2$ | Block |
|---|---|---|---|---|---|---|---|---|

**Supplementary Table S8. Factors Associated With Psychological Distress: PNI Vulnerability Model**

| Variables | *B* | *SE* | *β* | *t* | *p* | *95% CI for B* | $\Delta R^2$ | Block |
|---|---|---|---|---|---|---|---|---|
| **Demographic / Context Variables** | | | | | | | | |
| Age | -0,009 | 0,008 | -0,028 | -1,120 | 0,263 | [-0,024, 0,007] | 0,001 | 1 |
| Gender (Male vs Female) | -0,092 | 0,045 | -0,053 | -2,050 | 0,041 | [-0,179, -0,004] | 0,003 | 1 |
| Faculty (HSS vs FM) | 0,196 | 0,043 | 0,140 | 4,593 | <0,001 | [0,112, 0,279] | 0,013 | 1 |
| Faculty (CE vs FM) | 0,245 | 0,048 | 0,149 | 5,137 | <0,001 | [0,151, 0,338] | 0,016 | 1 |
| Relationship status (Single vs In a relationship) | -0,089 | 0,036 | -0,063 | -2,516 | 0,012 | [-0,159, -0,020] | 0,004 | 1 |
| Number of Family Members | 0,030 | 0,012 | 0,059 | 2,384 | 0,017 | [0,005, 0,054] | 0,003 | 1 |
| **Health / Trauma Variables** | | | | | | | | |
| Psychological help-seeking (Yes vs No) | 0,168 | 0,045 | 0,097 | 3,697 | <0,001 | [0,079, 0,257] | 0,008 | 2 |
| Trauma exposure (Yes vs No) | 0,111 | 0,038 | 0,079 | 2,928 | 0,004 | [0,037, 0,185] | 0,005 | 2 |
| Insomnia (Yes vs No) | 0,174 | 0,054 | 0,085 | 3,234 | 0,001 | [0,068, 0,280] | 0,006 | 2 |
| Chronic Fatigue (Yes vs No) | 0,304 | 0,038 | 0,216 | 7,983 | <0,001 | [0,230, 0,379] | 0,038 | 2 |
| **Relationship Functioning** | | | | | | | | |
| Family relationship quality (higher score = poorer relationship) | 0,122 | 0,023 | 0,137 | 5,313 | <0,001 | [0,077, 0,167] | 0,017 | 3 |
| **Personality** | | | | | | | | |
| PNI Vulnerability (mean of subscales) | 0,403 | 0,023 | 0,457 | 17,289 | <0,001 | [0,357, 0,448] | 0,179 | 4 |

**Model Summary**

$R^2$ = 0,510; Adjusted $R^2$ = 0,503; $\Delta R^2$ (Block 4) = 0,179; F change = 298,902 (df1=1, df2=820); p < 0,001

Block 1: F change (6; 827) = 12,29 p<,001

| Variables | *B* | *SE* | *β* | *t* | *p* | *95% CI for B* | Δ$R^2$ | Block |
|---|---|---|---|---|---|---|---|---|
| Block 2: F change (10; 823) = 36,37 p<,001 | | | | | | | | |
| Block 3: F change (11; 822) = 36,96 p<,001 | | | | | | | | |
| Block 4: F change(12; 821) = 71,08 p<,001 | | | | | | | | |

**Supplementary Table S9. Summary Table - Comparison of Regression Models Predicting Psychological Distress (BSI-GSI) Across PNI Operationalizations**

| Model including | $R^2$ | Adj. $R^2$ | Δ$R^2$ (Block 4) | F change | PNI predictor β |
|---|---|---|---|---|---|
| PNI Total | 0.551 | 0.544 | 0.219 | 400.871 | 0.508 |
| PNI Grandiosity | 0.562 | 0.556 | 0.230 | 431.336 | 0.524 |
| PNI Vulnerability | 0.510 | 0.503 | 0.179 | 298.902 | 0.457 |

*Note. Δ$R^2$ and F change refer to the incremental contribution of the PNI predictor entered in Block 4, after adjustment for demographic, family, health-related, and family relationship quality covariates entered in Blocks 1–3. All F changes significant at $p < .001$.*
